# Allergic Anisakiasis: An Integrated Review of Human, Animal and Cellular Evidence

**DOI:** 10.3390/biom16050648

**Published:** 2026-04-27

**Authors:** Stefania Isola, Emanuela Zumbo, Francesca Dimasi, Paola Lucia Minciullo, Sebastiano Gangemi

**Affiliations:** Unit and School of Allergy and Clinical Immunology, Department of Clinical and Experimental Medicine, University of Messina, 98125 Messina, Italy; stefania.isola@unime.it (S.I.); emanuela.zumbo.96@gmail.com (E.Z.); francescadimasi5@gmail.com (F.D.); sebastiano.gangemi@unime.it (S.G.)

**Keywords:** *Anisakis simplex*, anisakis allergy, gastro-allergic anisakiasis, IgE-mediated allergy, not IgE-mediated allergy, foodborne parasites, TRIF pathway, occupational exposure, in vitro models, public health

## Abstract

Allergic anisakiasis (AA), caused by the ingestion of fish contaminated with Anisakis larvae, has emerged as a growing global health concern due to the increasing consumption of raw or undercooked seafood. *Anisakis simplex* is identified as the primary etiologic species, responsible for gastrointestinal symptoms, IgE-mediated (Type I) or cell-mediated (Type IV) manifestations, and gastro-allergic anisakiasis (GAA), a unique clinical overlap between parasitic infection and acute IgE-mediated food allergy. In this review, we analyzed the epidemiology of *Anisakis simplex* allergy, the main diagnostic methods to confirm a diagnosis of food allergy, its clinical manifestations, and how these differ in different countries around the world. This multidisciplinary synthesis provides, for the first time, an integrated understanding of *Anisakis*-induced disease mechanisms across human, animal, and cellular levels. The persistence of allergenic proteins despite standard food processing underscores the need for improved diagnostic tools, public health surveillance, and preventive strategies—particularly in populations with high seafood consumption or occupational exposure. A comprehensive approach combining clinical, molecular, and immunological perspectives is essential to address the expanding global burden of allergic anisakiasis.

## 1. Introduction

Allergic anisakiasis (AA) represents a growing global public health concern, primarily due to the increasing popularity of consuming raw or undercooked fish. This review aims to summarize current knowledge on clinical manifestations, immunological mechanisms, diagnostics, and epidemiology of AA, highlighting emerging risk factors.

Anisakiasis, or Anisakidosis, is a human parasitic infection caused by the third-stage larvae of nematodes belonging to the genus *Anisakis*. These parasites have a marine life cycle. Humans are accidental hosts for nematode parasites that cannot progress their life cycles in humans. Live larvae generally penetrate the wall of the stomach or intestine, causing mainly gastrointestinal symptoms that, although they can be severe, usually last no more than two weeks due to the parasite’s death and/or expulsion. On the other hand, numerous cases of human sensitization to *Anisakis* are known as allergic anisakiasis (AA), which results in mild to severe allergic manifestations such as urticaria, angioedema, or anaphylaxis following the consumption of raw fish [1].

Diagnosis and treatment usually involve endoscopic extraction and identification of larvae. Allergic conditions are often diagnosed by prick-test and/or the detection of allergen-specific IgE and treated with an appropriate anti-allergy treatment. Furthermore, patients are advised to avoid future consumption of marine fish and cephalopods (such as squid), given the risk of *Anisakis* infection.

In 1988, a group of experts on the standardized nomenclature of animal parasitic diseases recommended the use of three different terms: anisakidosis for disease caused by any member of the family Anisakidae, anisakiasis for disease caused by members of the genus *Anisakis*, and pseudoterranovosis for disease caused by members of the genus Pseudoterranova [2,3].

The European Food Safety Authority considers *Anisakis* a major parasitological risk to humans associated with fish consumption, due to its ability to cause serious pathological conditions in humans as well as its global distribution [4].

To date, *Anisakis* species implicated in cases of human infection include A. simplex, *A. pegreffii* and *A. physeteris*, with the first two being responsible for the majority of reported cases [5,6]

In addition, several species belonging to the genera Phocanema (a recently resurrected genus encompassing five species formerly belonging to the genus Pseudoterranova) and Contracaecum, within the family Anisakidae, are also considered zoonotic and are recognized as causative agents of anisakiasis [7,8].

The first suspicion of *Anisakis*-like larvae in humans dates back to 1950, when they were found in the feces of an Inuk in Alaska. The first official case was reported in 1960 in the Netherlands by Van Thiel, who identified a “marine worm” within an eosinophilic granuloma in a patient with acute abdominal pain [9,10].

Anisakiasis is widespread throughout the world, with cases reported in 34 countries. The countries with the highest number of published cases are Japan, Spain, South Korea, Italy, and the United States [11].

In Southeast Asian countries (Philippines, Myanmar, Cambodia, Vietnam, Indonesia, Malaysia, Brunei) and the Pacific region, despite high fish consumption and the widespread presence of *Anisakis* larvae in edible fish [12,13], few or no clinical cases have been reported [14]. The same is true in Scandinavian countries where, despite fish being highly infected [15], there are few reports of infection in humans [11].

These observations suggest that factors other than raw fish consumption may influence susceptibility to infection [11].

Australia is considered the region with the highest *Anisakis* diversity, and there are numerous reports of infestation in edible fish [16].

The hypothesis of an association between allergic manifestations and acute parasitism put forward over 30 years ago [17] has subsequently been supported by both in vivo and in vitro tests for Anisakis, which have demonstrated the presence of an IgE-mediated mechanism [17].

## 2. Epidemiology

Since then, the rates of sensitization to *Anisakis* spp. in the general population and in specific occupational settings (mainly allergic patients and fishing industry workers) have increased globally, particularly in nations characterized by high seafood consumption such as Japan, Spain, South Korea, and Italy. Hypersensitivity prevalence estimates vary widely according to geographical area, characteristics of the population studied, diagnostic criteria and laboratory assays with varying sensitivity and specificity. Data from various areas with high fish consumption paint a diverse picture. In Galicia [18], the use of highly specific tests (capture ELISA) revealed a low level of IgE positivity (0.43%), closely linked to the consumption of homemade marinated anchovies (boquerones). In contrast, in Norway [19], the prevalence appears higher (up to 6.6% in atopic subjects), but the literature warns that these figures may be overestimated due to cross-reactivity with other tropomyosins present in shellfish and dust mites.

In emerging areas such as northern Morocco [20], immunoblotting and ImmunoCAP confirm a sensitization rate of 5.1% even in the absence of reported clinical cases, highlighting a “silent exposure” phase that precedes the epidemiological manifestation of the disease. In Catalonia [21], the Cibus Project has included Anisakis among the main causes of food allergies in adults (over 14 years), differentiating it from classic childhood allergies to milk and eggs and confirming the centrality of the Mediterranean diet in the dynamics of this hypersensitivity. A Japanese study [22] identified Anisakis as the main cause of anaphylaxis in coastal regions. In fact, in a group of patients who experienced an adverse reaction after ingesting fish, 91% of them tested positive for anisakis-specific antigens, rather than fish-specific antigens.

The wide variability in the prevalence of hypersensitivity is linked to various factors. A 2018 systematic review and meta-analysis involving eleven countries highlighted a wide range of sensitization percentages in the general asymptomatic population, depending on the diagnostic method used: in vivo tests (range 6.6–19.6%) or in vitro tests (0.4–27.4%). The percentage was higher in occupationally exposed workers. However, in patients with a clinical history of symptoms related to fish consumption, the prevalence of allergy ranged from 0 to 14% [23].

In Italy, prevalence ranges from 0.4% to 12.7%, with higher rates in the coastal areas of the Adriatic and Tyrrhenian Seas [24]. Southern regions show lower rates, while large northern cities have significantly higher rates than smaller towns. The main cause of sensitization is linked to the consumption of marinated anchovies. Conversely, where anchovies are typically fried, sensitization is lower. In cities, the increasing consumption of sushi and carpaccio also plays a role in the spread of sensitization.

In Sicily, sensitization to *Anisakis simplex* has been observed even in monosensitized subjects, confirming the importance of dietary habits [25].

Studies on occupationally exposed seafood workers have demonstrated an increased risk of sensitization to *Anisakis simplex*. An Italian study showed that fishermen and sailors had a higher prevalence of *Anisakis*-specific IgE compared to other fishing industry workers, with IgE levels tending to increase with the duration of exposure [23]. Consistently, earlier studies reported a significantly higher frequency of specific IgE positivity among fishermen and fishmongers compared with control populations [26].

Finally, a study conducted on 805 pediatric patients showed a sensitization prevalence of 6.1%, suggesting that *Anisakis* allergy represents a public health problem even among the pediatric population in Italy [27,28].

## 3. Methods

We conducted a comprehensive review of all studies on *Anisakis* to data, covering its global prevalence, clinical differences across countries, and experimental research involving cell and animal models.

The search was performed on PubMed using the following keywords: *Anisakis simplex*, *Anisakis* global prevalence, *Anisakis simplex* in humans, in vitro and in vivo studies AND Allergy, hypersensitivity, cell-mediated reaction, immune system.

Our analysis included all English-language articles published between 2000 and 2025. We included observational and retrospective studies, case reports and case series, while review articles were excluded. Particular attention was paid to data regarding the pathophysiology and clinical presentation of allergic reaction to *Anisakis* as well as the epidemiology of clinical manifestations.

## 4. Discussion

### 4.1. Immunological Mechanisms in Anisakis Simplex Allergy

Type I hypersensitivity

**Figure 1:**
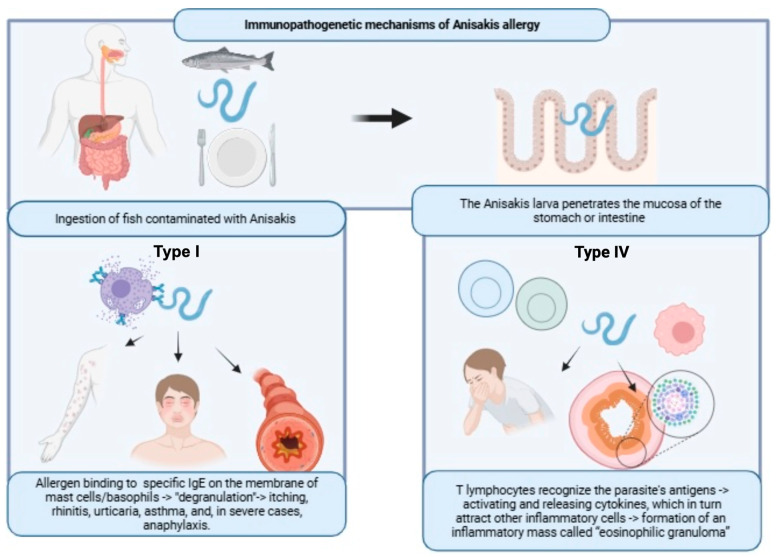
After ingestion of contaminated fish, Anisakis larva can penetrate gastric or intestinal mucosa and elicit an immunological mechanism. In a type I reaction (IgE-mediated or immediate mechanism), specific IgE antibodies—previously produced and bound to the membrane of mast cells and basophils—link the *Anisakis* allergen. This triggers the release of preformed mediators responsible for symptoms. In a type IV reaction (delayed or cell mediated mechanism), *Anisakis* allergens activate the lymphocytes, which release cytokines and chemokines. These, together with inflammatory cells, are responsible for the formation of eosinophilic granuloma. Created in BioRender. Dimasi, F. (2026) https://BioRender.com/dzl440z (accessed on 22 April 2026).

Allergy to *Anisakis simplex* is mainly associated with an immediate hypersensitivity mechanism known as Type I hypersensitivity, as shown in Figure 1.

When a susceptible individual comes into contact with an antigen, such as *A. simplex*, B lymphocytes are stimulated to produce IgE antibodies specific to that allergen. The specific IgE antibodies bind to high-affinity FcεRI receptors on mast cells and basophils (sensitization phase). Upon re-exposure to the same allergen, previously sensitized mast cells and basophils rapidly degranulate, releasing preformed mediators. The main substance released is histamine, but other inflammatory substances such as leukotrienes, prostaglandins, and cytokines are also liberated.

This degranulation results in symptoms such as itching, rhinitis, urticaria, asthma, and, in severe cases, anaphylaxis.

Type IV hypersensitivity

A delayed immunological mechanism has also been described in *Anisakis* allergy, which falls within the framework of Type IV (cell-mediated) hypersensitivity, with involvement of T-lymphocytes as shown in Figure 1 [29].

When the Anisakis larva penetrates the mucosa of the stomach or intestine, T lymphocytes recognize the parasite’s antigens and become activated. Once activated, they begin releasing cytokines, which in turn attract other inflammatory cells specifically eosinophils and macrophages, to the site of invasion. The accumulation and organization of these inflammatory cells around the larva and its antigens takes between 24 h and several days after ingestion.

This process results in the formation of an inflammatory mass called eosinophilic granuloma in the gastrointestinal wall. This lesion is frequently associated with symptoms such as abdominal pain, nausea, vomiting, and diarrhea [30,31].

### 4.2. Diagnostic

As a first step in diagnosing an *Anisakis* allergy, in addition to a careful medical history, the first test typically performed is the skin prick test (SPT). The allergens usually tested alongside *Anisakis* are food allergens (mainly fish such as tuna, salmon, mackerel, sardine, shrimp, octopus) and inhalants (mainly dust mites such as *Dermatophagoides pteronyssinus*, *Dermatophagoides farinae*, *Acarus siro*, *Lepidoglyphus destructor*, *and Tyrophagus putrescentiae*) to evaluate cross-reactivity.

If the SPT is negative but the clinical history strongly suggests an IgE-mediated reaction, the next step is to measure specific IgE antibodies through blood tests. *Anisakis*-specific IgE positivity has been shown to persist for many years, even more than eight years after the allergic episode [32]. Specific and total IgE levels are highly variable in the months following the allergic and parasite-specific reaction [33].

Allergen contamination of seafood plays a role in increasing *Anisakis*-specific IgE levels [32].

To date, 14 types of proteins (Ani s 1–14) have been identified as allergens of *A. simplex* (Table 1).

The main molecular components involved are the antigens Ani s 1 and Ani s 3. Ani s 3 is a tropomyosin molecule capable of cross-reacting with other tropomyosins, such as Der p 10 (antigen 10 of *Dermatophagoides pteronyssinus*) and Pen a 1 (antigen 1 of shrimp) [34].

Another example of tropomyosin cross-reactivity is the case of a patient who showed symptoms of anaphylaxis after ingestion of saury. This patient tested positive for *Anisakis*-specific IgE and SPT, but negative for saury prick-to-prick [35].

IgE antibodies against the Ani s 7 antigen can be induced only when the live larva injects it into the host tissues [36]. In another study, conducted on 12 patients through double-blind oral provocation tests, the results showed that only live larvae can induce allergic symptoms [37].

The study evaluated how the different clinical patterns varied in relation to positivity for Ani s 1 or Ani s 7. The positivity to Ani s 1 is mainly associated with gastro-allergic anisakiasis, while this molecule is not considered a major allergen in patients with chronic urticaria [38].

Recently, *Anisakis* hemoglobin has been described as a major allergen (Ani s 13) [39]. Additionally, an IgE-positive clone was isolated encoding a 23.5 kDa protein, named Ani s 14. Recombinant Ani s 14 was successfully expressed in *Escherichia coli* as a His-tagged protein and was shown to be IgE-reactive in 14 (54%) of 26 sera from patients allergic to *Anisakis* [40].

Ani s 5, Ani s 8 and Ani s 9, as shown in Table 1, belong to the SXP/RAL protein group, which cross-reacts with proteins from other parasites (e.g., *Ascaris lumbricoides*). Therefore, in areas with a high prevalence of intestinal parasites, positivity to these allergens may lead to an overestimation of *Anisakis* allergies.

It has been demonstrated that *Anisakis simplex* can also induce cell-mediated immune responses. When the reported symptoms are consistent with Type IV immune-mediated reactions, a patch test is indicated. In a study by Ventura et al., patch tests were positive in eight patients (80%) for live larvae, in five patients (50%) for frozen larvae, and in one patient (10%) for cooked larvae [29].

The Basophil Activation Test (BAT) has proven to be a highly effective tool in Anisakis allergy diagnosis, especially in regions with high seafood consumption [41]. The diagnostic efficacy of the conjunctival provocation test demonstrated a sensitivity of 75% and a specificity of 68.7%, with a positive predictive value of 67.7% and a negative predictive value of 75.9%. However, the authors conclude that, although there is a significant difference, the test is not accurate enough to be used as a standalone tool. In other words, it is not a perfect method for distinguishing those at risk for severe allergies from those who are not. Its diagnostic value, while present, is limited [42].

**Table 1:** Molecular allergens of *A. simplex*. Adapted from [43].

Molecular Allergens	Protein Categories	Biological Function
Ani s 1	Serin protease inhibitors like major species-specific allergen	Molecules indicating true sensitization to the parasite
Ani s 2	Paramyosin Cross reactivity with paramyosin from other nematodes and arthropods	Muscle-origin molecules that cross-react with similar molecules in other species
Ani s 3	Tropomyosin Extremely stable protein Shares epitopes with homologous proteins from sources such as house dust mites, food and insects	Muscle-origin molecules that cross-react with similar molecules in other species
Ani s 4	Cysteine protease inhibitor	Molecules indicating true sensitization to the parasite
Ani s 5	SXP/RAL-2 family protein	Molecules belonging to the SXP/RAL protein group which cross-react with proteins of other parasites
Ani s 6	Serine protease inhibitor	Minor allergen that shows a serine-type endopeptidase inhibitor activity
Ani s 7	139 kDa protein of unknown function The most important excretory Anisakis allergen	Molecules indicating true sensitization to the parasite
Ani s 8	SXP/RAL-2 family protein	Molecules belonging to the SXP/RAL protein group which cross-react with proteins of other parasites
Ani s 9	SXP-RAL-2 family protein	Molecules belonging to the SXP/RAL protein group which cross-react with proteins of other parasites
Ani s 10	21 kDa protein of unknown function	Protein that has no homology with any other protein
Ani s 11	27 kDa protein of unknown function	Not yet identified biological function
Ani s 12	31 kDa protein of unknown function Very stable protein; can trigger immune activation even after cooking	Not yet identified biological function
Ani s 13	Hemoglobin May contribute to delayed-onset or chronic allergic responses	Muscle-origin molecules that cross-react with similar molecules in other species
Ani s 14	24–27 kDa protein of unknown function	Molecules indicating true sensitization to the parasite

### 4.3. Manifestations

*Anisakis* spp. are known to cause gastrointestinal infections, which are typically categorized into acute, chronic, ectopic, or allergic manifestations (Table 2) [44].

In cases of gastric anisakiasis, there is a notable predilection for larval penetration along the greater curvature of the stomach, where the larvae embed themselves within the gastric wall. Endoscopic evaluation often reveals pronounced mucosal edema at the site of larval invasion [45].

The classic clinical presentation is characterized by acute, intense epigastric pain occurring within hours of ingesting raw or undercooked fish contaminated with Anisakis larvae. Symptom onset generally occurs within 12 h post-ingestion [46].

In contrast, intestinal anisakiasis tends to manifest with non-specific gastrointestinal symptoms such as nausea, vomiting, and diarrhea, typically developing within five days after exposure. It is well established that the intestinal form exhibits a longer latency period compared to its gastric counterpart [47].

Historically, the gastric subtype has accounted for approximately 95% of all reported anisakiasis cases, with the intestinal form comprising the remaining 5% [48].

However, intestinal anisakiasis is frequently underdiagnosed or misdiagnosed due to its clinical overlap with other gastrointestinal disorders, including inflammatory bowel disease, intestinal obstruction, peptic ulcer disease, acute appendicitis, diverticulitis, ileitis, and cholecystitis [49].

This diagnostic ambiguity likely contributes to the underreporting of intestinal cases.

*Anisakis simplex* is recognized as one of the most significant hidden food allergens, implicated in approximately 10% of previously unexplained idiopathic anaphylaxis cases and a substantial proportion of urticaria episodes in the adult population. In sensitized individuals, ingestion of Anisakis spp. triggers an IgE-mediated immune response, leading to a spectrum of clinical manifestations ranging from urticaria and angioedema to, in severe cases, severe anaphylaxis [50].

Acute allergic reactions typically occur within 15 to 30 min, though onset may be delayed up to 2–6 h following the consumption of contaminated fish. These reactions are more prevalent among adults aged 40 to 70 years and may range from mild gastrointestinal symptoms mimicking viral gastroenteritis to life-threatening anaphylactic shock requiring intensive care management [17].

The reported frequencies of clinical manifestations are consistent with other anaphylaxis cohorts, with cutaneous symptoms observed in nearly all patients (100%), respiratory involvement in 39%, and hypotension or syncope in 23%. Notably, gastrointestinal symptoms are also common, affecting approximately 74% of cases [17].

The clinical entity recognized as GAA occurs when the ingestion of raw or undercooked fish containing live Anisakis larvae results in both local gastrointestinal symptoms due to mucosal invasion and systemic allergic reactions [51,52].

Studies have demonstrated that intestinal permeability is significantly increased in patients with Anisakis allergy, particularly in those presenting with more severe clinical symptoms. Importantly, cessation of raw fish consumption for a period of six months has been associated with measurable improvement in intestinal barrier function [53].

Allergic reactions following the ingestion of seafood contaminated with *Anisakis* spp. or its antigens may be triggered by either somatic antigens or excretory–secretory (ES) products released by the infective larvae. To date, fourteen distinct allergens from *Anisakis simplex* (designated Ani s 1 through Ani s 14) have been identified, with the majority belonging to the ES protein group.

Serological analyses of blood samples from patients exhibiting allergic manifestations of anisakidosis have consistently demonstrated strong IgE reactivity to Ani s 1, Ani s 5, and Ani s 7—recognized as the principal allergens of the parasite. Ani s 1 has been detected in approximately 85% of individuals with clinical symptoms following prior parasitic exposure, while Ani s 7, an ES antigen, has been identified during the acute phase in 100% of patients with confirmed *Anisakis*-related allergy [3].

Importantly, thermal processing and freezing, although effective for devitalizing infective larvae, do not mitigate the allergenic potential of *Anisakis* spp. allergens. These proteins exhibit remarkable resistance to both high and low temperatures, rendering current food safety measures insufficient to fully prevent allergic reactions in sensitized individuals [3].

In a study conducted by Trujillo MJ et al., the risk of developing urticaria or angioedema was assessed across three dietary groups: one excluding fish entirely, one consuming fish frozen for 48 h, and one consuming fresh fish. In both fish-consuming groups, the fish was cooked prior to ingestion. The results indicated a significantly lower incidence of allergic symptoms in the group consuming frozen fish compared to the fresh fish group [54].

Similarly, Garcia demonstrated that patients with confirmed *A. simplex* allergy tolerated oral challenge tests with previously frozen fish containing dead larvae, without developing anaphylactic symptoms [55]

Another investigation evaluated the efficacy of a fish-free diet in 17 patients with positive SPT and specific IgE to *Anisakis*. The study reported reductions in both total and specific IgE levels, along with the absence of urticaria/angioedema episodes in compliant patients. However, a separate study found that *Anisakis*-specific IgE levels remained largely unchanged in the majority of participants (15 out of 19), with only three showing a decrease and one exhibiting a slight increase [56,57].

Beyond classical allergic presentations, eosinophilic inflammation has also been associated with *Anisakis*-induced immune responses. A case of eosinophilic esophagitis was documented in a patient presenting with dysphagia and recurrent urticaria, with a positive SPT for *Anisakis* [58].

In 2000, a boy with eosinophilic gastritis showed a type I hypersensitivity reaction to *Anisakis simplex*, confirmed through SPT, specific IgE quantification, and antigen-specific histamine release assays. Clinical remission was achieved following the elimination of fish and shellfish from the patient’s diet [59].

Cases of occupational allergy have been reported in Spain, Italy, and South Africa [17].

Rhinoconjunctivitis and occupational asthma caused by *Anisakis simplex* have been described in fishmongers, fishermen, processing factory workers, and farmers exposed to fishmeal and an isolated case involving a housewife. A recent review of occupational allergens includes this parasite as a rare cause of occupational reactions [17].

Protein contact dermatitis has been associated with this parasite in restaurant workers and fish handlers [17].

**Figure 2:**
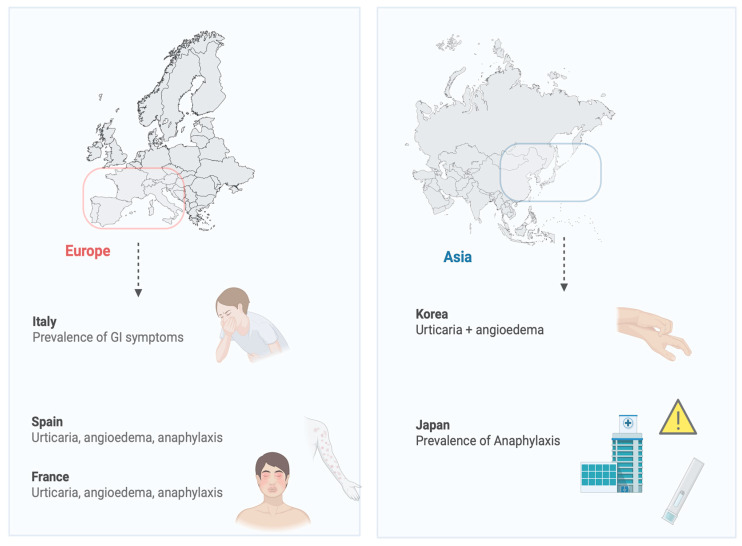
Different clinical manifestation around the world, mainly in European and Asian countries. In Italy, GI symptoms are more frequent, whereas in Spain and France urticaria, angioedema and anaphylaxis are the most common manifestations. Among Asian countries, acute and chronic urticaria and angioedema predominate in Korea, while anaphylaxis is more prevalent in Japan. Created in BioRender. Dimasi, F. (2025) https://BioRender.com/24h23nf (accessed on 22 April 2026).

Figure 2 depicts the global distribution of clinical symptom prevalence across different geographic regions.

In Spain, the clinical manifestations of allergy attributable to *Anisakis simplex* are consistently characterized by urticaria and/or angioedema. The cutaneous presentation is accompanied by gastrointestinal symptoms in 40% of cases and by anaphylactic shock in 12% [60].

Caballero et al. studied the clinical and immunological differences in Anisakis allergy between Italian and Spanish patients. The most frequently detected allergen among Italian and Spanish allergic patients was Ani s1. Patients suffering from gastrointestinal symptoms only were significantly more frequent among the Italians whereas the Spanish presented more frequently with urticaria, angioedema or anaphylaxis [61].

In Korea, patients diagnosed with *Anisakis* allergy most commonly present with acute or CU accompanied by angioedema. Approximately 30% of cases also exhibit abdominal pain and anaphylactic reactions. Notably, a significant proportion of affected individuals demonstrate an atopic background, frequently reporting a history of CU and food allergy, while allergic rhinitis appears less commonly [1].

A national retrospective survey was conducted in France to evaluate the incidence of anisakiasis. Of 37 patients, seven were confirmed cases with evidence of the worm, 12 were possible cases with abdominal pain after consuming raw fish and detected anti-Anisakis precipitins, and 18 were allergic, with acute manifestations after consuming fish, characterized by anisakiasis-specific IgE. This study demonstrates that approximately half of human anisakiasis cases are allergic, illustrating the emerging allergic potential of Anisakis [62].

In Japan, particularly in coastal regions with high fish consumption, *Anisakis* represents a significant cause of anaphylaxis, especially among adults. The frequent intake of raw fish markedly increases exposure to *Anisakis*-derived allergens [22].

Another Japanese study investigated the risk of recurrent hypersensitivity reactions and found that consuming raw seafood after being diagnosed with anisakis allergy increased the risk of hypersensitivity reactions in the study population. In contrast, ingesting cooked or frozen fish does not increase this risk. This phenomenon is linked to the presence of an antigen in the living larva, which is necessary to cause severe allergic symptoms [63]. Also, Spanish and Italian studies suggest that sensitization to Anisakis is caused by the ingestion of live larvae of the parasite, not by allergens present in the tissues of already dead or cooked fish [18,64].

### 4.4. The Role of Anisakis Infection in the Pathogenesis of Urticaria

Special emphasis should be placed on the role of *Anisakis simplex* in CU, which represents a significant diagnostic challenge due to its frequently subtle and overlapping clinical manifestations.

In patients diagnosed with CU, a markedly elevated rate of sensitization to *Anisakis simplex* has been observed—52.6% compared to the general population—highlighting a potential etiological factor [65].

Sensitization to *Anisakis simplex* in conjunction with sensitization to *Ascaris*, fish proteins, and aeroallergens, has been strongly correlated with an increased risk of urticaria.

Notably, *Anisakis simplex* sensitization alone was associated with a nearly fourfold increase in risk (odds ratio: 3.86). Crucially, this sensitization emerged as an independent risk factor for recurrent acute urticaria, even in the absence of co-sensitization to *Ascaris*, fish, or other environmental allergens [66]. These findings highlight the importance of *Anisakis simplex* as a significant and autonomous etiological factor in recurrent acute urticaria, providing valuable insight for both diagnosis and therapeutic management [66].

Further immunological characterizations revealed that immediate-onset urticaria is distinct from its prolonged counterpart. In a comparative study, researchers evaluated three patient cohorts—all sensitized to AS: those with GAA (urticaria < 48 h), those with prolonged acute urticaria (PROL; lasting from 3 days to 6 weeks), and a control group with chronic urticaria (CU+) [67].

Patients in the GAA group exhibited significantly elevated levels of total IgE, *Anisakis*-specific IgE, IgG, and IgG4 compared to both the PROL and CU+ groups. No statistically significant differences were noted between the PROL and CU+ groups in terms of these immunoglobulin profiles. These findings suggest that immediate urticaria may involve a more robust and distinct immunologic activation, differentiating it from other urticarial phenotypes within the spectrum of *Anisakis*-related hypersensitivity [67].

### 4.5. Murine Model-Based Analysis of Anisakis Pathophysiology

Experimental infection with third-stage larvae (L3) of *Anisakis simplex* in laboratory animals, particularly rats, represents a reliable model for investigating the immunological and allergic mechanisms underlying human anisakiasis [68].

As early as 2003, Sánchez-Monsálvez et al. reported that both viable and digested (non-viable) larvae can modulate autonomic regulation of intestinal motility via cholinergic and adrenergic pathways, suggesting that such effects may precede or occur independently of IgE-mediated responses. Their study employed short-term infection protocols and in vitro exposure to crude parasite extracts (CE) to analyze motility alterations across duodenum (RD), jejunum (RJ), and ileum (RI). The results revealed dose- and time-dependent hyperreactivity to carbachol and blockade of noradrenaline in tissues exposed to increasing L3 concentrations, with selective muscarinic hyperreactivity observed in RD and generalized adrenergic suppression enhancing transit exclusively in RD. Conversely, CE exposure elicited a dose-dependent increase in smooth muscle tone and contractile amplitude, most pronounced in RI > RJ > RD—an inverse distribution compared to active infection [69].

Further investigation into Anisakis-induced allergic manifestations has focused on occupational exposures, particularly the emergence of contact dermatitis among fishery workers. Murine models, including BALB/c wild-type and genetically modified strains deficient in IL-4, IL-13, or IL-4Rα signaling pathways, were subjected to epicutaneous sensitization with repeated topical applications of Anisakis extract. This protocol induced local skin inflammation, epidermal hyperplasia, TH2 cytokine production, and the generation of parasite-specific IgE and IgG1 antibodies. Intravenous challenge subsequently triggered systemic anaphylaxis, which was markedly reduced in mice lacking IL-4 or with impaired IL-4Rα signaling in CD4^+^ T cells, highlighting the differential contributions of these pathways to cutaneous and systemic allergic responses [70].

Despite advances in understanding adaptive immunity in anisakiasis, the role of innate immune mechanisms remains less defined. Recent studies isolating excretory–secretory (ES) proteins from L3 demonstrate that intranasal administration in mice markedly increases IL-17 expression in pulmonary tissues and regional lymph nodes.

Primary epithelial and MEF cells exposed to ES proteins upregulated IL-6 and CXCL1 levels, a response abrogated in TRIF-deficient (TRIF^−^/^−^) MEF cells, indicating reliance on TRIF-dependent innate signaling [71]. Notably, RNase treatment of ES proteins did not mitigate the inflammatory cascade, excluding dsRNA involvement. These findings underscore the capacity of Anisakis-derived ES proteins to evoke innate pulmonary inflammation via non-viral, TRIF-mediated pathways, expanding our understanding of helminth-induced immune modulation [71].

### 4.6. Exploring the Pathogenicity of Anisakis spp. Through In Vitro Models

**Figure 3:**
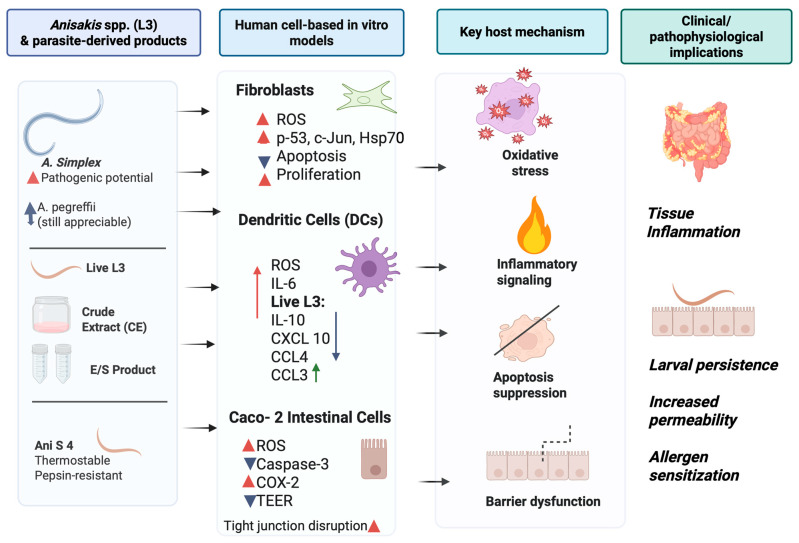
Pathogenicity of Anisakis spp. through in vitro models. Human fibroblasts treated with Anisakis CE and ES products led to upregulation of reactive oxygen species, inhibition of apoptosis and cellular proliferation. Human dendritic cells exposed to live L3 or CE showed different profiles, with upregulation of certain cytokines and chemokines secretion and downregulation of others. The Caco-2 cells exposed to CE caused increased oxidative stress and suppression of apoptosis. The red and green arrow and red triangle indicate an increase, the blue arrow and triangle indicate a decrease, and the black arrow indicates the effect. Created in BioRender. Dimasi, F. (2026) https://BioRender.com/diux20t (accessed on 22 April 2026).

In vitro models have been employed to investigate the pathogenic potential of L3, particularly in relation to cellular oxidative stress, immunological activation, and inflammatory responses during anisakiasis. A schematic representation summarizing the main pathogenic mechanisms described in this section, including oxidative stress, immunological activation, and inflammatory responses, is provided in Figure 3.

The ability of L3 to penetrate host tissues—whether in natural or accidental hosts—and to survive in gastric juice is considered a key indicator of pathogenicity. Several studies have explored these traits across *Anisakis* species, revealing interspecies differences in virulence. Notably, four independent investigations demonstrated that *A. simplex* s.s. exhibits greater pathogenic potential, evidenced by higher penetration rates in fish filets and agar, as well as enhanced survival in artificial gastric juice. In contrast, *A. pegreffii* showed lower but still appreciable pathogenic capacity [72,73]. Pathogenicity may also be influenced by the host cellular response following exposure to live larvae or parasite-derived components such as CE and ES products. However, culturing parasitic nematodes remains technically challenging, and current in vitro and in vivo models often fail to replicate the human physiological context that characterizes natural infection. Despite these limitations, human-derived cell cultures have been utilized to explore host–parasite interactions.

Studies involving non-immune cells, such as human fibroblasts, have provided insights into larval migratory behavior and extracellular matrix disruption, given the role of fibroblasts in connective tissue synthesis. Treatment with CE and ES products led to an upregulation of reactive oxygen species (ROS), p53, c-jun, c-fos, and Hsp70, promoting inflammation and inhibiting apoptosis—thereby favoring cellular proliferation [74].

The immunomodulatory effects of *Anisakis* products were further examined using human dendritic cells (DCs), which play a central role in antigen presentation and the transition from innate to adaptive immunity. Upon exposure to live L3 or CE, immature and mature DCs exhibited differential cytokine and chemokine secretion profiles. Both treatments induced IL-6 expression; however, only live L3 significantly downregulated IL-10, CXCL10, CCL4, and ICAM, while upregulating CCL3. CE-treated cells showed a mild, non-significant decrease in these markers. Additionally, both conditions impaired DCs’ viability and maturation, as evidenced by reduced expression of HLA-DR, CD86, CD83, and CCR7, and elevated ROS levels. When autologous CD4+ T cells were stimulated with DCs exposed to L3 or CE, they failed to produce IL-4, IL-17, and IL-10, suggesting a hyporesponsive DC phenotype insufficient to elicit a Th2/Th17 immune response [75].

The Caco-2 cell line, derived from human colorectal adenocarcinoma, is widely used as a model of the intestinal epithelial barrier due to its ability to form monolayers with characteristics akin to small intestinal enterocytes. This model has proven valuable for studying intestinal parasitic infections and inflammatory mechanisms. Exposure to *Anisakis* CE resulted in increased oxidative stress, suppression of apoptosis-related markers, and induction of inflammatory pathways [76].

Specifically, CE treatment led to a marked reduction in caspase-3 activation and a significant increase in COX-2 expression, indicating that *Anisakis* may modulate key cellular processes involved in inflammation, proliferation, and cell death.

More recently, the Caco-2 model was used to assess the impact of *A. simplex* CE on intestinal integrity and permeability, with a focus on the Ani s 4 allergen [77].

Researchers observed a decrease in transepithelial electrical resistance following CE exposure that was not attributable to cytotoxicity or protease activity. This compromised barrier function was linked to elevated ROS production and altered localization of tight junction proteins. Furthermore, the thermostable Ani s 4 antigen, resistant to pepsin digestion, was shown to translocate across the intestinal epithelium to the basolateral side, a phenomenon previously described for other food allergens.

## 5. Limitations of the Study

This review is based exclusively on the literature retrieved from PubMed and adopts a narrative synthesis approach. The included evidence derives mainly from observational and retrospective studies, as well as case reports, often conducted on small sample sizes and highly selected populations. These study designs are inherently prone to selection bias and frequently lack appropriate control groups. Therefore, findings have been interpreted with caution, emphasizing consistent patterns across studies rather than drawing definitive conclusions.

A substantial proportion of the literature originates from countries with high consumption of raw or undercooked fish (e.g., Spain, Japan, South Korea). This may limit the generalizability of findings to populations with different dietary habits, genetic backgrounds, and environmental exposures. Geographical differences have been highlighted, and overgeneralization of epidemiological and clinical data has been avoided.

Persistent elevation of specific IgE levels, cross-reactivity with other allergens (e.g., crustaceans, house dust mites, storage mites), and variability in confirmatory diagnostic strategies—including the limited feasibility of oral food challenge tests—further complicate interpretation. These aspects are critically discussed to underline diagnostic uncertainties and study heterogeneity.

Experimental data from murine and in vitro models have provided insights into the immunopathogenic mechanisms of *Anisakis* spp. However, these models do not fully replicate human immune complexity and fail to account for host-specific genetic, environmental, and dietary factors. Consequently, the translational applicability of preclinical findings is limited. In this review, experimental findings are integrated with clinical evidence, with clear distinction between mechanistic hypotheses and clinically validated data.

## 6. Conclusions and Future Perspectives

In conclusion, Anisakis is a significant and growing public health concern, particularly in regions with a high consumption of raw or undercooked seafood.

While its presence has been documented for decades, the increase in global culinary trends, such as the popularity of sushi and other raw fish dishes, has led to a rise in both gastrointestinal and allergic forms of anisakiasis. Countries with the highest consumption of fish products, including Japan, Spain, South Korea, and Italy, have the highest incidences. Anisakis not only causes gastrointestinal infections but is also responsible for symptoms such as urticaria, angioedema, anaphylaxis, and acute respiratory distress syndrome. Furthermore, some studies have suggested a possible correlation with chronic urticaria.

The diagnostic process for Anisakis allergy is complex, requiring a combination of careful patient history, blood tests for total and specific IgE, and SPT, the first-line tests due to their high sensitivity. However, their specificity is drastically reduced by molecular cross-reactivity that leads to false positives in patients sensitized to other nematodes (such as Ascaris lumbricoides, in case of positivity to Ani s 5, Ani s 8, and Ani s 9) or arthropods (house dust mites and crustaceans, in case of positivity to Ani s 3). To overcome these limitations, molecular diagnostics (Component-Resolved Diagnosis, CRD) emerges as the most accurate tool for risk stratification. While positivity to Ani s 1 and Ani s 7 is a highly specific indicator of true sensitization (with Ani s 7 linked to exposure to live larvae), reactivity to Ani s 3 signals pan-allergic cross-reactivity that often does not correlate with clinical symptoms after fish ingestion. A critical element for patient management is the identification of Ani s 4: as a thermostable and pepsin-resistant protein, its presence indicates a persistent risk of anaphylaxis even after consumption of properly cooked or frozen fish, unlike thermolabile allergens. In complex diagnostic scenarios, the Basophil Activation Test (BAT) offers superior specificity compared to sIgE, as it mimics the in vivo response and allows the distinction between persistent immunological memory (sIgE can remain positive for over 8 years) and a clinically active allergy. Finally, although provocative tests (oral or conjunctival) remain the theoretical reference for definitive confirmation, their poor standardization and the risk of serious adverse reactions limit their routine use.

The risk of sensitization to Anisakis is not only linked to dietary habits but also to professional exposure, as observed among fishermen and fish handlers. These occupational risks highlight the need for greater awareness and the implementation of protective measures and food safety protocols, such as industrial freezing, which effectively eliminates the larvae.

Studies in animal and cellular models have shown that the parasite not only acts through IgE; it actively alters the intestinal epithelial barrier, increasing its permeability and triggering oxidative stress and systemic inflammation through innate immune pathways (such as the TRIF-dependent pathway).

Overall, the spread of anisakiasis underscores the need for continued research, public health education campaigns, and stricter regulations in the seafood industry to prevent infections and allergic reactions. This is crucial for protecting both consumers and those in the seafood supply chain, especially in light of changing dietary patterns and the ongoing challenges of parasite control in a globalized food market.

## Figures and Tables

**Table 2:** Clinical presentations of Anisakis infection.

	Clinical Manifestation	Onset Time	Diagnostic Test
Gastric anisakiasis	Epigastric pain Nausea, vomiting	Within a few hours up to 12–24 h after ingestion of raw or undercooked fish	Upper gastrointestinal endoscopy Dietary history
Intestinal anisakiasis	Abdominal pain Diarrhea	1–7 days	Abdominal CT scan Ultrasound
IgE-mediated allergic reaction	Urticaria, dyspnea Hypotension Anaphylaxis	Minutes to a few hours	Anisakis-specific igE Skin prick test

## Data Availability

No new data were created.
